# Laparoscopic versus open catheter placement in peritoneal dialysis patients: a systematic review and meta-analysis

**DOI:** 10.1186/1471-2369-13-69

**Published:** 2012-07-27

**Authors:** Haiying Xie, Wei Zhang, Jun Cheng, Qiang He

**Affiliations:** 1 Kidney Disease Center, the First Affiliated Hospital, College of Medicine, Zhejiang University, Hangzhou, China; 2 The Nephrology Department, Shaoxing People's Hospital, Shaoxing, China

**Keywords:** Laparoscopic catheter placement, Peritoneal dialysis, Complications

## Abstract

**Background:**

Peritoneal dialysis has been proven to be a safe and effective mode of renal replacement therapy for patients with end-stage renal disease. The usage of laparoscopic catheter placement technique was increased in recent years. But the advantages and disadvantages between the laparoscopic catheter placement technique and open laparotomy technique were still http://in controversy. The objective of this study is to access the operation-related data and complications of catheter placement for peritoneal dialysis (PD) patients, Then to determine the better method for catheter insertion.

**Methods:**

We performed a systematic review and meta-analysis on published studies identified by the databases PubMed, EMBASE, Highwire, and the Cochrane Library. Analysis was performed using the statistical software Review Manager Version 5.0.

**Results:**

We assessed the operation-related data and complications of four randomized controlled trials (RCTs) and ten observational studies. The available data showed that laparoscope prolonged the time for catheter insertion in PD patients, however, the two groups did not significantly differ in hospital stays, early and late complications, including infection, dialysate leaks, catheter migration, pericannular bleeding, blockage and hernia.

**Conclusions:**

The data showed that Laparoscopic catheter placement had no superiority to open surgery. However, this treatment still needs to be confirmed in a large, multi-center, well-designed RCT.

## Background

Peritoneal dialysis has been proven to be a safe and effective mode of renal replacement therapy for patients with end-stage renal disease. Catheter placement, first developed in 1968 [1], was thought to be the key to successful peritoneal dialysis ( PD). Various techniques have been described for catheter insertion. Traditionally, an open laparotomy technique has been used via a lower abdominal incision. Recently, this technique had been used in catheter insertion in PD patients because of the development of the laparoscope. Laparoscopic guidance has been used to locate catheter under direct vision. Ash et al (1981) [2] first used peritonoscopy for guidance of peritoneal dialysis catheterization, and Jwo SC et al extended this technique to laparoscopy [3-17]. However, both methods carried their own risks, including exit infection or peritonitis, outflow obstruction, leakage and so on [18,19]. The ideal method of PD catheters insertion remained debatable. Quite a few research papers compared the advantages and disadvantages between these two surgical methods and suggested different conclusions [3-16]. Several authors [4,7,10,11,13,15] found a benefit by the addition of laparoscopic guidance, while the others [3,6,8,16] showed they were equivalent in complications and catheter survival. Because of this argument, we performed a meta-analysis of four randomized controlled trials (RCTs) and a systematic review of ten observational studies to compare laparoscopy with open placement of peritoneal dialysis catheter (PDC).

## Methods

### Search strategy

We conducted a search on PubMed, EMBASE, Highwire, and the Cochrane Library from 1990 using the keywords "laparoscopy", "laparoscopic", "catheter" and "peritoneal dialysis". All RCTs and observational studies comparing these two surgical methods were identified. There were no language restrictions on inclusion for the systematic review. We also used the "related articles" function to broaden the search. All abstracts, studies, and citations scanned were reviewed. References of the articles acquired were also searched by hand. The latest date for this search was October 20, 2011.

### Inclusion criteria

We included all RCTs and observational studies which compared the laparoscopic catheter implantation with open technique and reported the surgical complications, operation-related data, and other relevant information. The studies should have been published as full-length articles in peer-reviewed journals.

### Exclusion criteria

We excluded studies if the surgical complications, operation-related data, and other relevant information were not clearly reported; or it was impossible to extractor to calculate the appropriate data from the published results. Duplicate publications were also excluded.

### Data extraction

We extracted data from the RCTs and observational studies included in terms of patient characteristics, sample size,, mean age, gender, history of abdominal surgery, and the following reported outcomes:(1)The operation time, duration of hospital stay,(2)The incident rate of catheter -related complication such as infection, dialysate leak, catheter migration, outflow obstruction, pericannular bleeding, blockage and hernia. Data extraction was performed by two independent reviewers (H.X. and J.C.). Disagreements between these two investigators regarding studies included were solved by consensus.

### Quality assessment

We access the methodological quality of observational studies using the Newcastle–Ottawa scale [20]. A quality score was calculated on the basis of three major components: selection of the groups of study (0 to 4 points), comparability of groups (0 to 2 points), and assessment of outcome (0 to 3 points). The maximum score is 9 points, representing the highest methodological quality.

We assessed the methodological quality of RCTs using adapted criteria from the Cochrane Handbook version 5.0.1 from the Cochrane Collaboration guidelines [21]. Assessing risk of bias: sequence generation; allocation concealment; blinding of participants and personnel; blinding of outcome assessment; incomplete outcome data; selective outcome reporting; other potential sources of bias.

### Statistical analysis

We carried out the meta-analysis using Review Manager Software (RevMan, version 5.0) provided by the Cochrane Collaboration [22]. For continuous outcomes, we expressed the results using the weighted mean difference (WMD) with 95% confidence intervals (CIs). For dichotomous outcomes, we planned to report results as odds ratio (OR) with 95% CIs. We accessed the statistical heterogeneity between studies using the χ² test and evaluated the extent of inconsistency using the I² statistic. A fixed-effect model was used for calculation of summary estimates and their 95% CI unless they had significant heterogeneity, in which case results were confirmed using a random-effect statistical model. P < 0.05 was considered statistically significant. We conducted separate analysis for observational studies and RCTs.

## Results

### Search results

**Figure 1:**
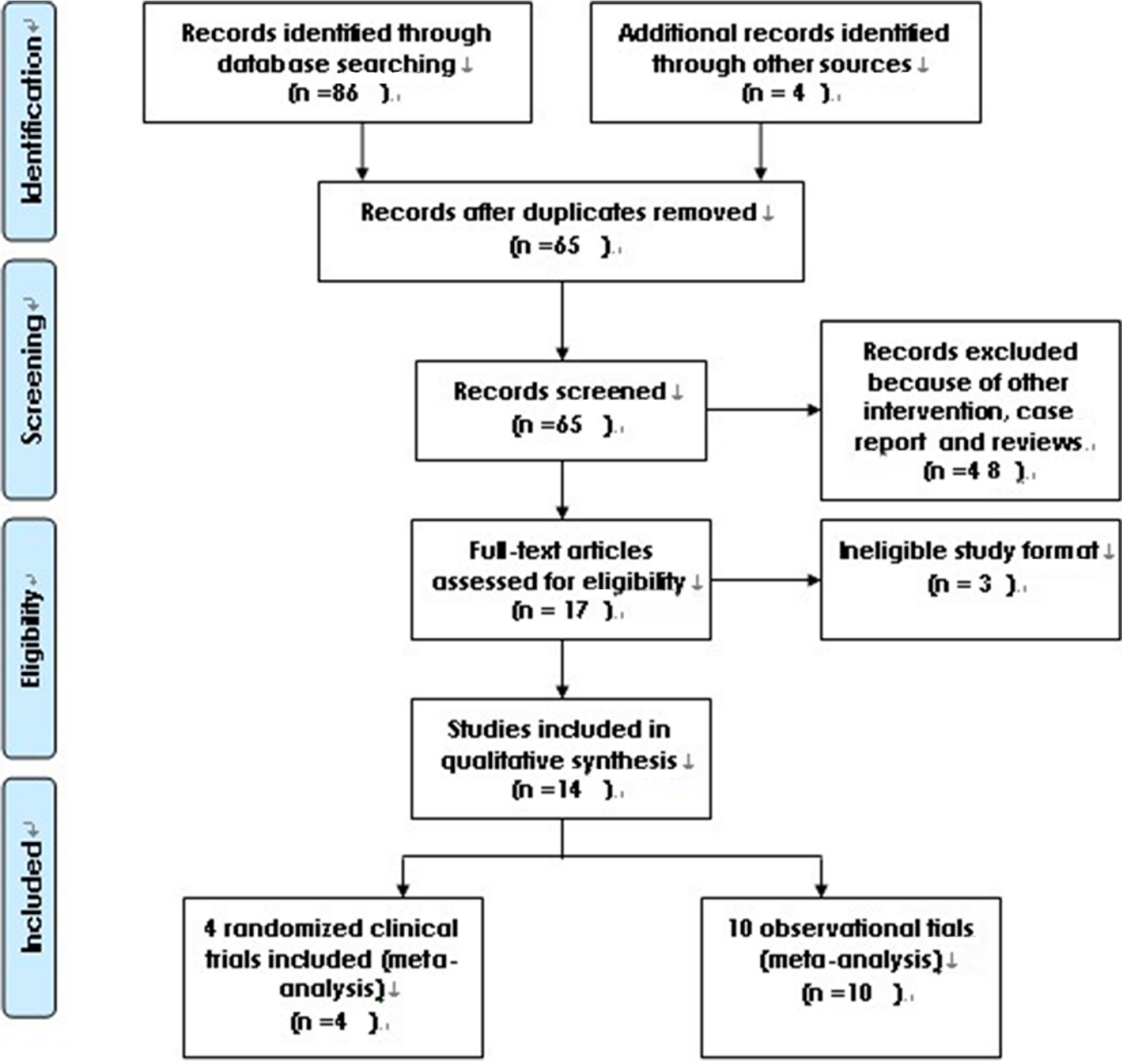
**Flow chart for the selection**.

As shown in Figure 1, the search strategy identified four randomized controlled trials [3-6] and ten observational studies [7-16] that compared the results of laparoscopic to open surgical method for peritoneal dialysis catheter implantation. All the studies were published in English. Publication dates ranged from 1998 to 2010. Table 1 contains specific information on study design, sample size, age, sex, history of prior abdominal surgery and outcome in this systematic review.

**Table 1:** Basic imformations of the studies

**Ref**	**Cases**		**Age**		**Sex (male/female)**		**History of prior abdominal surgery**		**Operativing time**		**Hospital stays**		**Outcome**
	**Laparoscopic group**	**Open group**	**Laparoscopic group**	**Open group**	**Laparoscopic group**	**Open group**	**Laparoscopic group**	**Open group**	**Laparoscopic group**	**Open group**	**Laparoscopic group**	**Open group**	
Jwo SC 2010 [3]	37	40	56.65 ± 13.99	54.43 ± 16.49	12/25	18/22	4	5	68.32±31.90	46.68 ± 15.99	14.81 ± 5.61	13.08 ± 6.80	complications
Tsimoyiannis EC 2000 [4]	25	25	58(25–74)	62(48–78)	18/7	16/4	——	——	29 ± 7	22 ± 5			complications
Gadallah MF 1999 [5]	76	72	45 ± 1.8	47.2 ± 2.4	37/39	22/34	37	33					complications
Wright MJ 1999 [6]	21	24	46.4 ± 14.8	49.3 ± 20.2	14/7	15/9	11	5	21.8 ± 2.9	14.3 ± 3.3	3.1 ± 1.9	2.4 ± 0.8	complications
Gajjar AH 2007 [7]	45	30	——	——	——	——	14	4					complications
Mattioli G 2007 [8]	17	17	14.2(1.4-20.5)	13.4(1.1-17.3)	8/7	7/7	3	2	55(30-115)	50(30-65)			complications
Soontrapornchai P 2005 [9]	50	52	55 ± 11	60 ± 11	33/17	35/17	——	——	65 ± 17	29 ± 3			complications
Crabtree JH 2005 [10]	78	63	55.8 ± 13.1	49.5 ± 13.7	42/36	38/25	43	19					complications
	200		54.4+14.3	——	108/92	——	106	——					
Oğünç G 2003 [11]	21	21	51.1 ± 2.0	44.2 ± 3.6	12/9	8/13	11	0	45.4 ± 5.1	30.9 ± 1.3	1.1 ± 0.1	3.1 ± 0.6	complications
Daschner M 2002 [12]	25	23	6.9(2 m-19.3Y)	3.2(2d-19.2Y)	——	——	——	——	21.8 ± 2.9	14.3 ± 3.3	3.1 ± 1.9	2.4 ± 0.8	complications
Batey CA 2002 [13]	14	12	48.9(20–72)	46(19–62)	8/6	9/3	——	——	41.7 ± 12.742	55.7 ± 14.285	0.14	1.5	complications
Crabtree JH 2000 [14]	150	63	55 ± 13.4	49.5 ± 13.7	85	38	35	19					complications
Draganic B 1998 [15]	30	30	56.5(19-74)	63.2(18-83)	16/14	7/22	15	13					complications
Eklund B 1998 [16]	65	43	50.4(27-75)	52.6(20-75)	34/26	22/20	——	——					complications

[3-6] is RCTs, [7-16] is observational studies.

### Study quality

The quality of the observational studies varied from 5 to 7 points, with a mean of 6.5 points. Most studies were of moderate to high quality.

Sequence allocation concealment was only used in two of the included RCTs [3,4,6]. Blinding was unclear in either of the included RCTs, except for one study [6]. All the RCTs were analyzed on incomplete outcome data or selective outcome reporting. Other potential sources of bias are unclear. The studies were of low to moderate quality.

### Outcome

#### Observational studies

The results of operation-related data in observational studies were shown in Table 2. From Table 2, we found that laparoscope prolonged the time necessary for catheter insertion, but that there were no significant differences in hospital stays.

**Table 2:** The results of operation-related data and complications in observational studies

**Laparoscope vs open group**	**No. of studies**	***P*for Heterogeneity**	**Effect model**	**Pooled estimate**	**95% Confidence interval**	***P*for Test for overall effect**
operating times	4	< 0.00001	random	11.97	0.87 to 23.06	0.03
hospital stays	2	< 0.00001	random	-0.68	-3.33 to 1.96	0.61
exit site and tunnel infection	6	0.79	fixed	0.76	0.45 to 1.28	0.30
peritonitis	6	0.23	fixed	0.74	0.48 to 1.15	0.18
dialysate leaks	10	0.33	fixed	1.00	0.56 to 1.80	0.99
catheter migration	3	0.06	fixed	0.56	0.20 to 1.62	0.29
pericannular bleeding	3	0.22	fixed	0.40	0.09 to 1.85	0.24
blockage	7	0.04	random	0.66	0.27 to 1.62	0.36
hernia	3	0.72	fixed	1.10	0.47 to 2.58	0.83

The results of complications in observational studies were shown in Table 2. From Table 2, we found that that there were no significant differences in exit site and tunnel infection, peritonitis, dialysate leaks, catheter migration, pericannular bleeding, blockage and hernia between the two groups.

#### RCTs

The results of operation-related data in RCTs were shown in Table 3. From Table 3, we found that laparoscope prolonged the time necessary for catheter insertion, but that there were no significant differences in hospital stays.

**Table 3:** The results of operation-related data and complications in RCTs

**Laparoscope vs open group**	**No. of studies**	***P*for Heterogeneity**	**Effect model**	**Pooled estimate**	**95% Confidence interval**	***P*for Test for overall effect**
operating times	3	0.05	random	8.66	4.78 to 12.54	<0.0001
hospital stays	2	0.49	fixed	0.79	-0.04 to 1.63	0.06
infection	4	0.21	fixed	0.92	0.61 to 1.39	0.70
dialysate leaks	4	0.02	random	0.45	0.06 to 3.34	0.43
catheter migration	4	0.16	fixed	0.61	0.32 to 1.17	0.14
pericannular bleeding	2	0.96	fixed	3.35	0.92 to 12.24	0.07
hernia	2	0.65	fixed	1.55	0.25 to 9.46	0.63

The results of complications in RCTs were shown in Table 3. From Table 3, we found that there were no significant differences in complications of infection, dialysate leaks, catheter migration, pericannular bleeding and hernia between the two groups.

##### Subgroup analysis

For the main complications such as catheter-related infection, dialysate leak and catheter migration, early and late complications were mentioned in three [3,5,6] of the four RCTs. Subgroup analysis was used to reduce the heterogeneity in the study.

##### Infection

**Figure 2:**
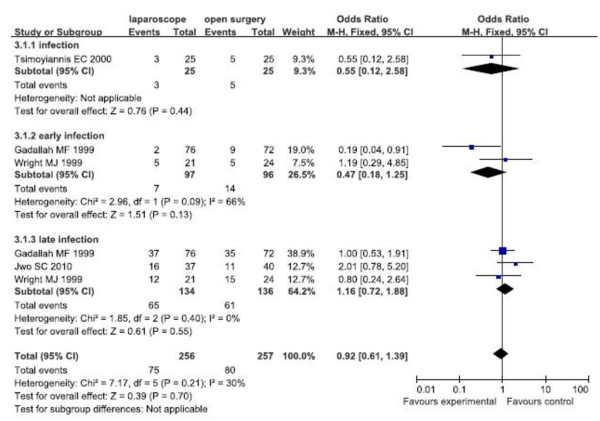
Subgroup analysis for infection of RCTs

The fixed effects model was used because there was no significant heterogeneity. Forest plots displayed the results of the meta-analysis for early infection (OR = 0.47, 95% CI: 0.18,1.25; p = 0.13), late infection (OR = 1.16, 95% CI: 0.72,1.88; p = 0.55), overall infection (OR = 0.92, 95% CI: 0.61,1.39; p = 0.70). The results showed that there were no significant differences of early, late and overall infection between the two groups (Figure 2).

##### Dialysate leaks

**Figure 3:**
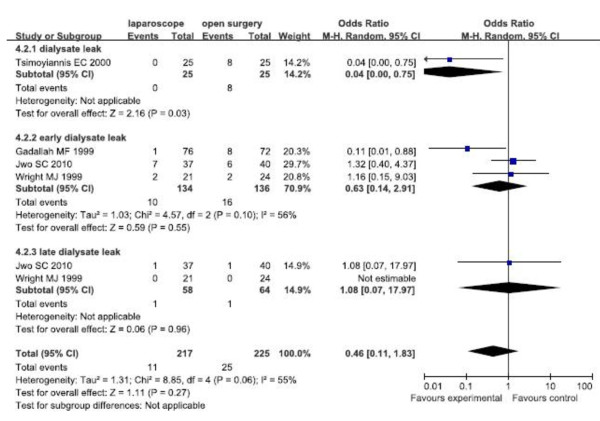
Subgroup analysis for dialysate leak of RCTs.

The random effects model was used because of the moderate heterogeneity of dialysate leak. Forest plots displayed the results of the meta-analysis for early dialysate leaks (OR = 0.63, 95% CI: 0.14,2.91; p = 0.55), late dialysate leaks (OR = 1.08, 95% CI: 0.07,17.97; p = 0.96), and overall dialysate leaks (OR = 0.46, 95% CI: 0.11,1.83; p = 0.27). The results showed that there were no significant differences of early, late and overall dialysate leaks between the two groups (Figure 3).

##### Catheter migration

**Figure 4:**
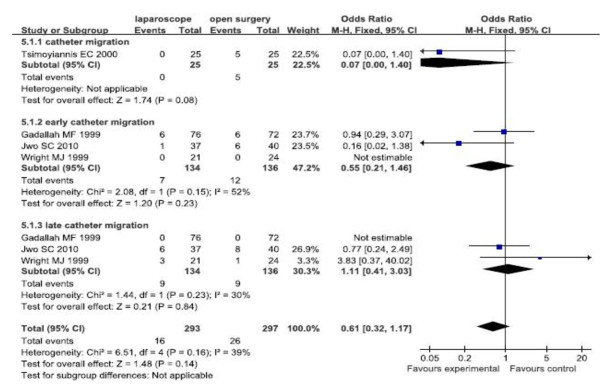
Subgroup analysis for catheter migration of RCTs.

The fixed effects model was used because there was no significant heterogeneity. Forest plots displayed the results of the meta-analysis for early catheter migration (OR = 0.55, 95% CI: 0.21,1.46; p = 0.23), late catheter migration (OR = 1.11, 95% CI: 0.41,3.03; p = 0.84), and overall infection (OR = 0.61, 95% CI: 0.32,1.17; p = 0.14). The results showed that there were no significant differences of early, late and overall catheter migration between the two groups (Figure 4).

## Discussion

In the present study, the results of the observational studies were consistent with that of RCTs. Forest plots showed that laparoscope prolonged the time necessary to carry out catheter insertion in PD patients. The two groups did not significantly differ in hospital stays, early and late complications, including infection, dialysate leaks, catheter migration, pericannular bleeding, blockage, and hernia. The laparoscopic group had a lower incidence of complications than that of the open group, but the differences did not reach statistical significance.

A few articles about the two techniques had already been published. Recently, John H. Crabtree made a review about the use of the laparoscope for dialysis catheter implantation, and provided us some suggestions for catheter placement [23]. Later, Jwo SC conducted a prospective randomized study for comparison of open surgery with laparoscopic-assisted placement of Tenckhoff peritoneal dialysis catheter and written a literature review, concluding that Laparoscopic assisted percutaneous puncture exhibited no superiority to open surgery [3]. His point of view was consistent with ours. So far, no meta-analysis had been made to compare the two methods. Therefore we made a meta-analysis, in order to make clinicians convenient to select the appropriate surgical approach. In our study, we included all the studies mentioned in two the reviews above and also searched other database, and analyze both RCTs and observational studies, and we drew a relatively clear conclusion that open surgery had the shorter operative time but similar effect to laparoscope. Laparoscopy was seemed not to reduce the incidence of catheter-related complication rates. However, it could allow for the rescue of blocked catheters [8]. It allowed immediate start dialysis without fluid leakage and permitted simultaneous performance of other laparoscopic procedures [4]. It provided the patient reduced perioperative discomfort and earlier return to full mobility [14]. It also allowed the diagnosis and treatment of the accompanying surgical pathologies during the same operation, such as intra-abdominal adhesions or preformed inguinal hernias [11,12]. Compared to traditional peritoneal dialysis catheter placement, laparoscopic catheter placement has smaller scar, less pain, and quicker recovery. This approach is safe, feasible, and completely visible. Dialysis tube can be fixed under laparoscope, and the catheter position is more precise. Laparoscopic catheter placement is also suitable for patients with a history of abdominal surgery or with abdominal adhesions. Omentum can be fixed and trimmed, and postoperative complications may be reduced under laparoscopy. The above advantages induced the interest of clinicians on the laparoscopic approach. On the other hand, this approach has potential problems including advanced technique, high cost, and relatively high anesthesia risk. Weighing the pros and cons, which surgical approach to choose depends on the specific conditions and clinicians.

The heterogeneity of some variables in this study is worthy of comment. Explanations included the following. The heterogeneity of the time for operation depended on the skill and experience of surgeon and different operative methods. The heterogeneity of the length of stay at hospital might caused by different standards for discharge at different hospitals. The heterogeneity of the dialysate leaks, and blockage might due to the operations by different surgeons, the different study designs, catheter types, and operation techniques. What is more, patients in one of the observational study were children [12], and it would affect the stability of results. In order to reduce the heterogeneity, we conducted a further research, and made subgroup analysis, but got the same conclusion. Moreover, Crabtree et al. divided laparoscopic catheterization into basic laparoscopy and advanced laparoscopy. The complications in Crabtree showed that mechanical flow obstruction was 1 in 200 implantation procedures in the advanced group, which was significantly less than that in the open dissection and basic laparoscopic groups [10]. So we classified the complications in basic laparoscopy and advanced laparoscopy and made subgroup analysis. The random-effects statistical model revealed significant heterogeneity. The results also showed that laparoscopy did not reduce complications.

Patients in the two groups were given antibiotic prophylaxsis in most of the studies [3,5-8,10-12]. Postoperative antibiotics were not prescribed only in one study [13]. Use of prophylactic antibiotics before catheterization was found to be effective in reducing procedure-related peritonitis [24]. Strippoli et al. stated that the use of perioperative intravenous antibiotics, compared to no treatment, significantly reduced the risk of early peritonitis [25]. The routine use of vancomycin for prophylaxis before catheterization is not recommended because of the emergence of vancomycin-resistant enterococci [26]. Other antibiotics, such as a cephalosporin, should be the first choice.

This study has several limitations. First, in observational studies, the proportion of patients who had previous abdominal surgery in laparoscopic group was larger than that of open group. More patients had chosen laparoscopy in observational studies [7,8,10,11,15], because laparoscopy offered the advantage of entrance to abdominal cavity under direct visualization, and it was superior to open surgery for patients with a history of abdominal surgery. This would have impact on the outcomes. Moreover, as mentioned in the studies, the technique and condition of performance were of wide variability. The inevitable consequence of these practice traits was that there were almost as many laparoscopic techniques for placing catheters as there were surgeons performing them [3]. In addition, the small number of participants, as well as the low quality of the studies, might not allow a reliable conclusion. All these factors can produce high selection bias, performance bias and measuring bias. Therefore, the studies in this review are limited, further trials of large-scale, high-quality RCTs are needed to find potential advantages or disadvantages.

## Conclusions

Open surgery needs a shorter operative time and simpler equipment requirement but has a similar effect to laparoscope. Therefore, the present study shows that laparoscopic catheter placement has no superiority to open surgery. But it is only a short-term outcome, further trials that focus on long-term outcomes are needed. The technology of laparoscopic catheter placement has been developping rapidly at present. Laparoscopic catheter placement can be done by the single-hole under local anesthesia now, with smaller scar and risk. In the future, advanced laparoscopy using more sophisticated procedures may reduce complications in catheterization.

## Competing interests

The authors have declared that no competing interests exist.

## Authors’ contributions

Conceived and designed the experiments: HX QH. Performed the experiments: HX QH WZ JC. Analyzed the data: HX QH. Wrote the first draft of the manuscript: HX . Contributed to the writing of the manuscript: HX JC ZW QH. ICMJE criteria for authorship read and met: HX JC ZW QH. Agree with manuscript results and conclusions: HX ZW JC QH. All authors read and approved the final manuscript.

## Pre-publication history

The pre-publication history for this paper can be accessed here:

http://www.biomedcentral.com/1471-2369/13/69/prepub
